# Nuclear cathepsin D enhances TRPS1 transcriptional repressor function to regulate cell cycle progression and transformation in human breast cancer cells

**DOI:** 10.18632/oncotarget.4394

**Published:** 2015-06-27

**Authors:** Anne-Sophie Bach, Danielle Derocq, Valérie Laurent-Matha, Philippe Montcourrier, Salwa Salwa Sebti, Béatrice Orsetti, Charles Theillet, Céline Gongora, Sophie Pattingre, Eva Ibing, Pascal Roger, Laetitia K. Linares, Thomas Reinheckel, Guillaume Meurice, Frank J. Kaiser, Christian Gespach, Emmanuelle Liaudet-Coopman

**Affiliations:** ^1^ IRCM, Institut de Recherche en Cancérologie de Montpellier, Montpellier, France; ^2^ INSERM U1194, Montpellier, France; ^3^ Université de Montpellier, Montpellier, France; ^4^ Institut Régional du Cancer de Montpellier, Montpellier, France; ^5^ Universität zu Lübeck, Lübeck, Germany; ^6^ Department of Pathology, CHU Nimes, Nimes, France; ^7^ Institute of Molecular Medicine and Cell Research, Albert-Ludwigs-University, Freiburg, Germany; ^8^ Functional Genomic Plateform, Institut Gustave Roussy, Villejuif, France; ^9^ INSERM U938, Molecular and Clinical Oncology, Paris 6 University Pierre et Marie Curie, Hôpital Saint-Antoine, Paris, France

**Keywords:** GATA-factor, BAT3, *PTHrP* promoter, yeast-two hybrid, confocal microscopy

## Abstract

The lysosomal protease cathepsin D (Cath-D) is overproduced in breast cancer cells (BCC) and supports tumor growth and metastasis formation. Here, we describe the mechanism whereby Cath-D is accumulated in the nucleus of ERα-positive (ER^+^) BCC. We identified TRPS1 (tricho-rhino-phalangeal-syndrome 1), a repressor of GATA-mediated transcription, and BAT3 (Scythe/BAG6), a nucleo-cytoplasmic shuttling chaperone protein, as new Cath-D-interacting nuclear proteins. Cath-D binds to BAT3 in ER^+^ BCC and they partially co-localize at the surface of lysosomes and in the nucleus. BAT3 silencing inhibits Cath-D accumulation in the nucleus, indicating that Cath-D nuclear targeting is controlled by BAT3. Fully mature Cath-D also binds to full-length TRPS1 and they co-localize in the nucleus of ER^+^ BCC where they are associated with chromatin. Using the LexA-VP16 fusion co-activator reporter assay, we then show that Cath-D acts as a transcriptional repressor, independently of its catalytic activity. Moreover, microarray analysis of BCC in which Cath-D and/or TRPS1 expression were silenced indicated that Cath-D enhances TRPS1-mediated repression of several TRPS1-regulated genes implicated in carcinogenesis, including *PTHrP*, a canonical TRPS1 gene target. In addition, co-silencing of TRPS1 and Cath-D in BCC affects the transcription of cell cycle, proliferation and transformation genes, and impairs cell cycle progression and soft agar colony formation. These findings indicate that Cath-D acts as a nuclear transcriptional cofactor of TRPS1 to regulate ER^+^ BCC proliferation and transformation in a non-proteolytic manner.

## INTRODUCTION

Cathepsins were originally identified as lysosomal proteases, but recent work highlighted their atypical roles in the extracellular space, cytoplasm and nucleus [1]. Cathepsin D (Cath-D) is one of the most abundant lysosomal endoproteinases implicated in protein catabolism. Human Cath-D is synthesized as a 52-kDa precursor that is converted to an active 48-kDa single-chain intermediate within endosomes and then to the fully active mature protease, which consists of a 34-kDa heavy chain and a 14-kDa light chain, in lysosomes. Cath-D catalytic site includes two critical aspartic residues (Asp 33 and 231).

Cath-D is also an independent marker of poor prognosis for breast cancer associated with metastasis [2, 3]. Indeed, Cath-D is overproduced by breast cancer cells (BCC) and the pro-enzyme is abundantly secreted in the tumor microenvironment [4]. Cath-D stimulates BCC proliferation, fibroblast outgrowth, angiogenesis, breast tumor growth and metastasis formation [5–12]. Secreted Cath-D enhances proteolysis in the breast tumor microenvironment by degrading the cysteine cathepsin inhibitor cystatin C [13] and promotes mammary fibroblast outgrowth by binding to LDL receptor-related protein-1 (LRP1) [14].

To better understand the mechanisms underlying Cath-D pro-tumoral activity, we carried out a yeast two-hybrid screening using the 48-kDa Cath-D form as bait and identified the nuclear proteins tricho-rhino-phalangeal-syndrome type 1 (TRPS1) and BAT3 as two Cath-D molecular partners. TRPS1, a multi zinc-finger nuclear protein, is an atypical GATA-type transcription repressor that binds to GATA sites on its target genes [15]. TRPS1 affects cell proliferation, differentiation and apoptosis essentially in bone and cartilage [16–22] and it overexpressed in breast cancer [23]. Recently, it was shown that in BCC, TRPS1 is inversely associated with the epithelial-to-mesenchymal transition (EMT) [24] and controls cell cycle progression and cell proliferation [25]. The nucleo-cytoplasmic shuttling protein BAT3 (known as Scythe/BAG6) controls apoptosis [26], DNA damage response [27], autophagy [28] and quality control of nascent peptides [29] in mammalian cells. We then investigated the nuclear role of Cath-D and its two partners in BCC homeostasis. We found that the chaperone BAT3 promotes Cath-D accumulation in the nucleus of ERα-positive (ER^+^), well-differentiated luminal epithelial BCC, where fully-mature Cath-D co-localizes with full-length TRPS1. Using a reporter gene assay, we demonstrate that Cath-D acts as a transcriptional repressor, independently of its catalytic activity, and enhances TRPS1 transcriptional repressor function. The transcriptional network controlled together by Cath-D and TRPS1 is required for cell cycle progression and maintenance of the transformed phenotype in luminal ER^+^ BCC.

## RESULTS

### Cath-D binds directly to the transcriptional repressor TRPS1 *in vitro*

**Figure 1:**
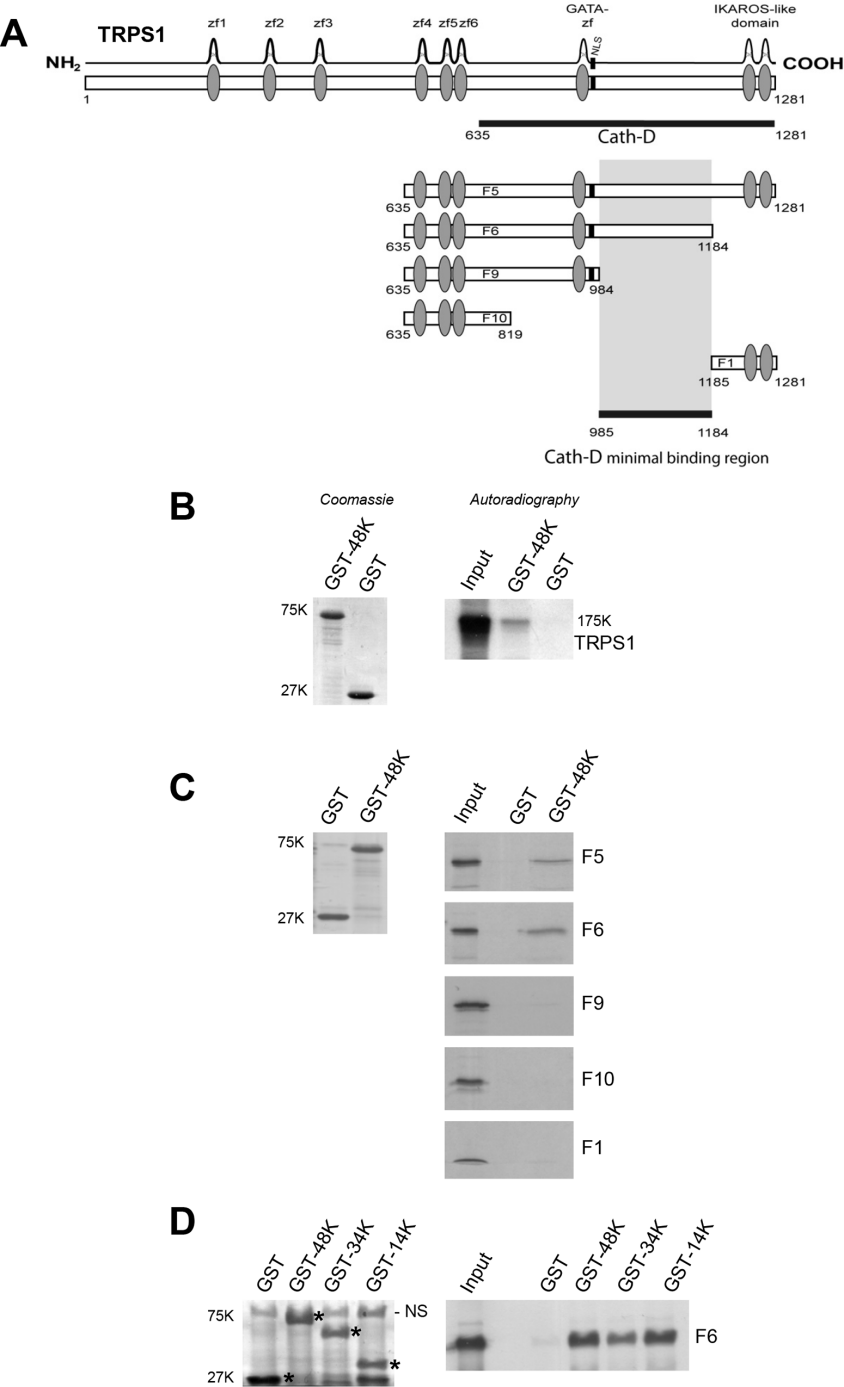
Cath-D binds to TRPS1 *in vitro* **A.** TRPS1 minimal region required for binding to Cath-D. TRPS1 (1281 aa) has nine putative zinc finger motifs. The seventh zinc finger is very similar to the GATA-type DNA-binding zinc fingers. TRPS1 also includes an IKAROS-like double zinc finger dimerization domain that mediates TRPS1 repressive function and a nuclear localization signal (NLS). The TRPS1 clone (residues 635–1281) identified in the yeast two-hybrid screen with 48-kDa Cath-D as bait is shown (black bar). Different TRPS1 fragments (F5, F6, F9, F10 and F1) were used for *in vitro* GST pull-down assays to determine the minimal region (aa 985–1184) required for binding to Cath-D. **B.** Binding of full-length TRPS1 to GST-48kDa Cath-D by GST pull-down. Radio-labeled full-length TRPS1 synthesized in a reticulocyte lysate system was incubated with glutathione-Sepharose beads containing GST-48K Cath-D or GST. GST proteins stained with Coomassie blue are shown in the left panel. Bound TRPS1 was detected by autoradiography (right panel). Input corresponds to 1/10 of the lysate used for the pull-down assay. K, molecular mass in kiloDaltons. **C.** Binding of TRPS1 fragments to 48-kDa Cath-D-GST. Radio-labeled TRPS1 fragments were incubated with beads containing GST-48K Cath-D or GST. GST proteins stained with Coomassie blue are shown in the left panel. Bound TRPS1 was detected by autoradiography (right panels). **D.** The F6 fragment of TRPS1 binds to the different Cath-D-GST forms. Radio-labeled F6 TRPS1 was incubated with beads containing GST-48K, 34K and 14K Cath-D forms, or GST. *GST proteins stained with Coomassie blue are shown in left panel. Bound F6 was detected by autoradiography (right panel). NS, non-specific

We identified Cath-D binding proteins by yeast two-hybrid screening of a randomly-primed BCC cDNA library using as bait the 48-kDa Cath-D form fused to the LexA DNA-binding domain. The clone (isolated in duplicate) encoded a 647-residue peptide that included the C-terminal region (aa 635–1281) of TRPS1 (Fig. 1A). We verified the direct binding of full-length TRPS1 to Cath-D by GST pull-down assays (Fig. 1B). TRPS1 fragments F5 (aa 635–1281) and F6 (635–1184) bound to 48-kDa GST-Cath-D, whereas F9 (aa 635–984), F10 (aa 635–819) and F1 (aa 1185–1281) did not (Fig. 1A and 1C). This narrowed the minimal Cath-D-binding site to a TRPS1 region of 200 aa (985–1184) that lies between the GATA DNA-binding zinc finger and the C-terminal IKAROS-like double zinc finger. Further analyses showed that TRPS1 fragment F6 bound also to both the 34-kDa heavy chain and the 14-kDa light chain of GST-Cath-D (Fig. 1D), indicating that the interaction interface involves both subunits of mature Cath-D.

### ER expression and EMT differentially affect Cath-D and TRPS1 expression in BCC

**Figure 2:**
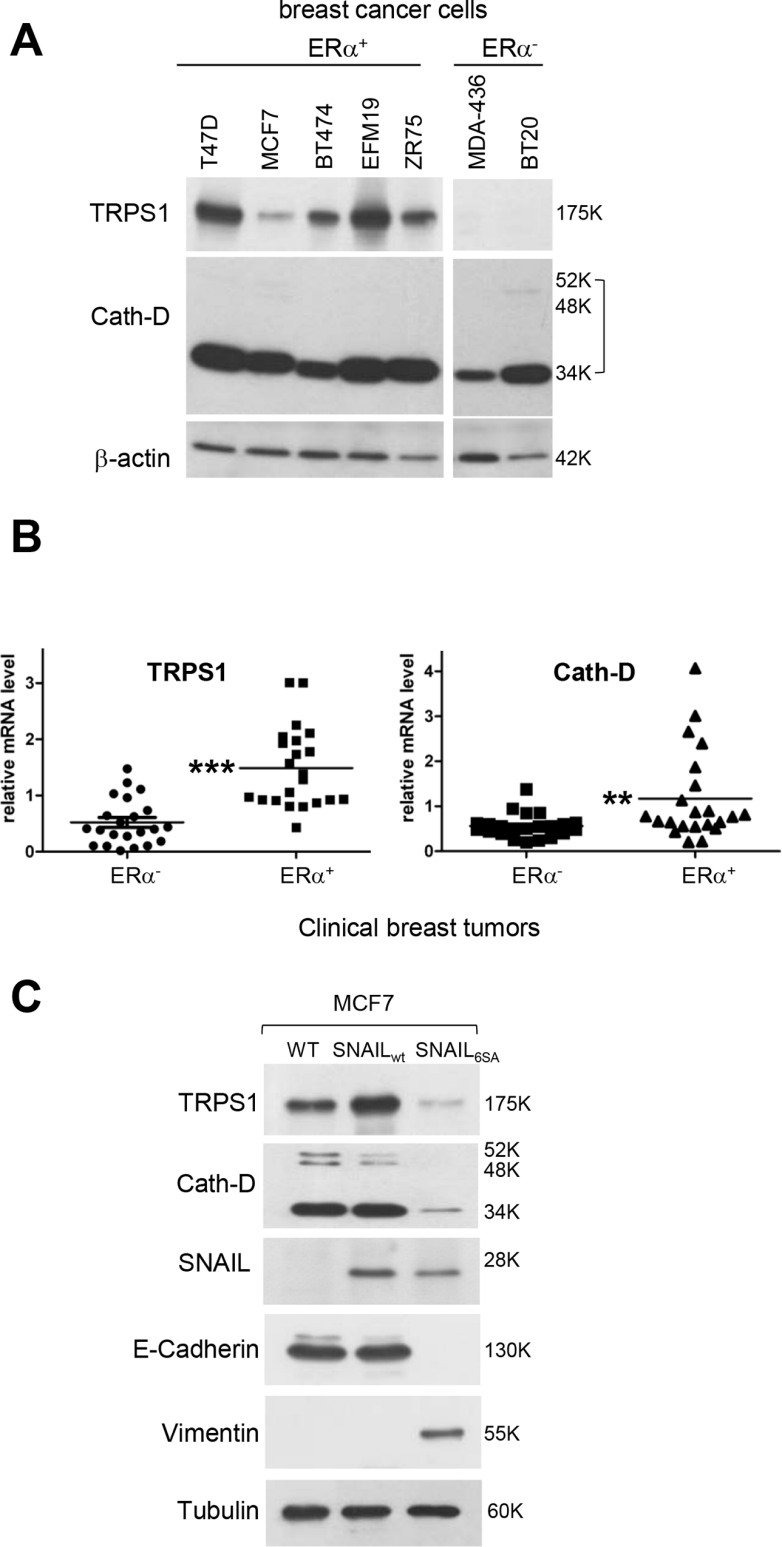
The ER status and EMT influence Cath-D and TRPS1 expression in human BCC lines and breast tumors **A.** Cath-D and TRPS1 expression in ER^+^ and ER^−^ BCC lines. Whole cell extracts (10 μg proteins) were separated by SDS-PAGE and analyzed by immunoblotting with anti-TRPS1 and Cath-D antibodies. β-actin: loading control. **B.** TRPS1 and Cath-D expression in ER^+^ and ER^−^ human breast tumor samples. TRPS1 and Cath-D mRNA levels were quantified in 22 ER^+^ and 22 ER^−^ breast tumor biopsies by RT-qPCR. Median ± SD of triplicate PCR assays. ***p < 0.0001 for TRPS1 and **p < 0.01 for Cath-D, Mann-Whitney *U*-test. **C.** Effect of EMT on Cath-D and TRPS1 expression in ER^+^ MCF7 cells. Cath-D, TRPS1, Snail, E-cadherin, vimentin and α-tubulin expression were assessed by immunoblotting in wild type MCF7 cells (WT) and MCF7 cells stably transfected with SNAIL WT or SNAIL-6SA (to induce EMT).

We then investigated Cath-D and TRPS1 expression in ER^+^/ER^−^ BCC lines and breast tumor samples (Fig. 2A and 2B). Cath-D was abundantly expressed in both ER^+^ and ER^−^ BCC lines and tumor samples, although it was up-regulated in ER^+^ tumors compared to ER^−^ tumors. TRPS1 was also significantly up-regulated in ER^+^ tumors compared to ER^−^ samples (Fig. 2B), but was only detected in ER^+^ BCC lines (Fig. 2A). The absence of TRPS1 expression in ER^−^ BCC lines could be related to its significant down-regulation in ER^−^ breast cancer samples; however, this finding needs to be validated in a wider number of ER^−^ BCC lines and by quantitative immunohistochemistry analysis. Differently from *CTSD*, the gene encoding Cath-D [30], estradiol did not further stimulate TRPS1 mRNA expression in ER^+^ MCF7 cells, suggesting that the *TRPS1* gene is not estradiol-dependent (Fig. S1). The ER^+^ BCC lines that express both Cath-D and TRPS1 were derived from luminal-like cancer subtypes with a more differentiated epithelial-like phenotype, frequently associated with the absence of EMT [31, 32]. We thus analyzed the effect of EMT induction on TRPS1 and Cath-D expression in ER^+^ MCF7 cells that were stably transfected with a Snail variant (6SA) that cannot be phosphorylated by GSK-3 beta and thus induces EMT [33]. As expected, E-Cadherin was down-regulated and vimentin was induced, indicating the occurrence of canonical EMT in 6SA-transfected MCF7 cells compared to controls (wild type Snail or untransfected cells). Conversely, both TRPS1 and Cath-D were repressed in these cells (Fig. 2C). Thus TRPS1 and Cath-D are co-expressed in ER^+^ well-differentiated luminal epithelial BCC under the negative control of EMT.

### Nuclear localization of Cath-D and TRPS1 in ER^+^ BCC

**Figure 3:**
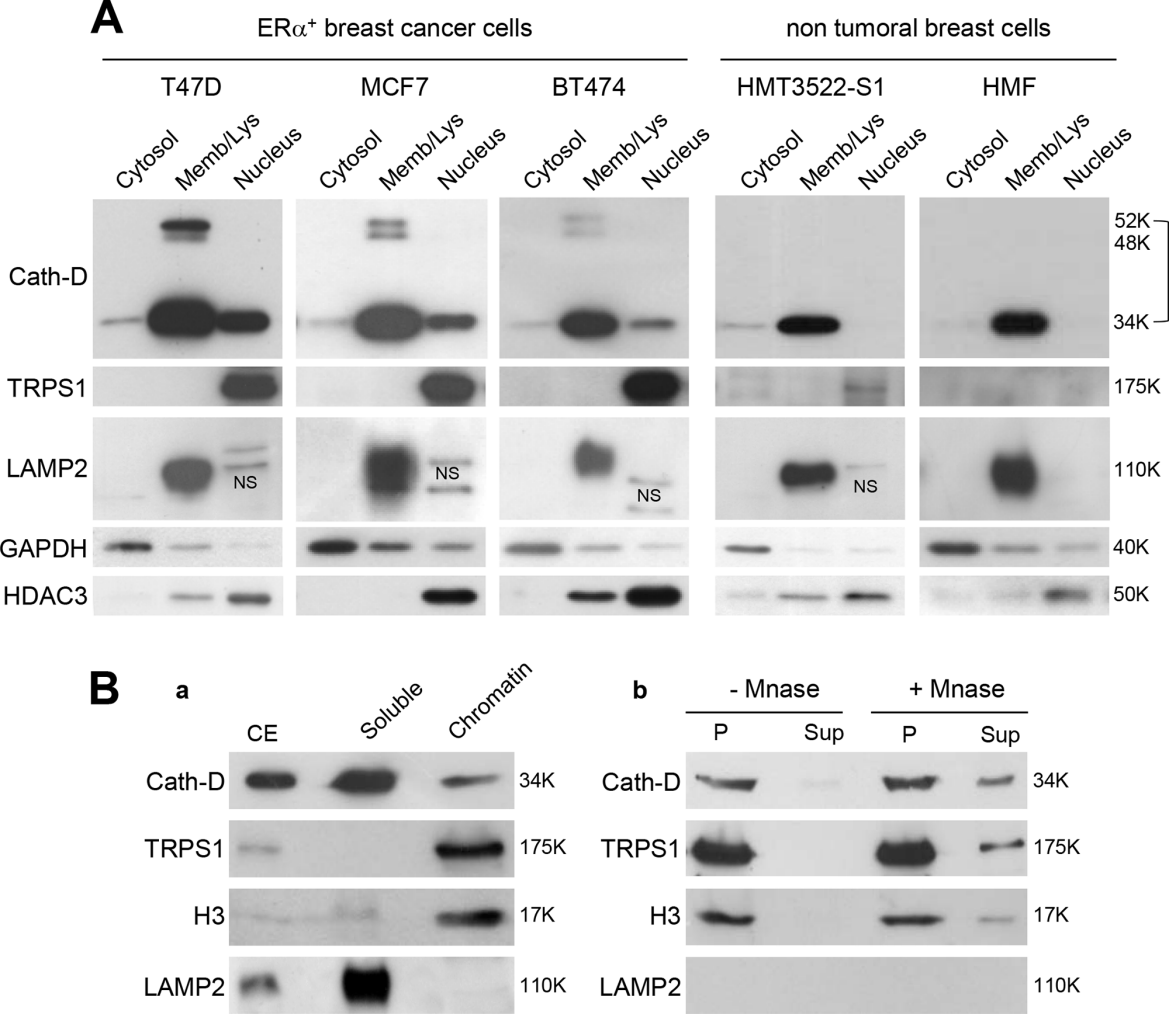
Nuclear localization of Cath-D and TRPS1 **A.** Subcellular localization of Cath-D and TRPS1. Cytoplasmic, membrane and nuclear fractions (10 μg) of different BCC lines (T47D, MCF7 and BT474), immortalized epithelial breast cells (HMT3522-S1) and human mammary fibroblasts (HMF) were analyzed by western blotting (WB) using antibodies against Cath-D, TRPS1, LAMP2 (marker of lysosomes), GAPDH (cytoplasm marker) and HDAC3 (nucleus). NS, not specific. **B.** Cath-D and TRPS1 in the chromatin-enriched fraction. Aliquots (10 μg) of cell extracts (CE), soluble and chromatin-bound fractions were separated by SDS-PAGE. Cath-D, TRPS1, histone H3 and LAMP2 were detected by WB. The lysosomal marker was LAMP2 and the chromatin-bound marker was Histone H3 (panel a). Chromatin-bound proteins (P) were treated (or not) with micrococcal nuclease (MNase). DNA and the associated proteins were collected in the supernatant (Sup) and analyzed by WB (panel b).

As TRPS1 is a nuclear protein [15], we examined the cellular localization of TRPS1 and Cath-D in ER^+^ BCC lines (T47D, MCF7 and BT474), immortalized human breast epithelial cells (HMT3522-S1) and human breast fibroblasts (HMF) (Fig. 3A). The 52-kDa, 48-kDa and 34-kDa forms of Cath-D were detected mainly in the membrane fraction that contains endosomes and lysosomes, together with the lysosomal marker LAMP2. However, a significant amount of mature Cath-D (34-kDa) was also in the nuclear fraction of the assessed ER^+^ BCC lines, together with full-length TRPS1 (175-kDa) and histone deacetylase 3 (HDAC3). Only very low levels of TRPS1 were detected in HMT-3522-S1 nuclear fraction, whereas both Cath-D and TRPS1 were not observed in HMF nuclei (Fig. 3A). Finally, both TRPS1 and Cath-D were detected in the chromatin-enriched fraction of T47D cells with histone H3, but not LAMP2 (Fig. 3B, panel a). TRPS1, Cath-D and H3 solubilization from the nucleus by micrococcal nuclease (Mnase) (Fig. 3B, panel b) indicated that TRPS1 and Cath-D are associated with chromatin and not with other insoluble structures.

### Nuclear interaction and co-localization of endogenous mature Cath-D and full-length TRPS1

**Figure 4:**
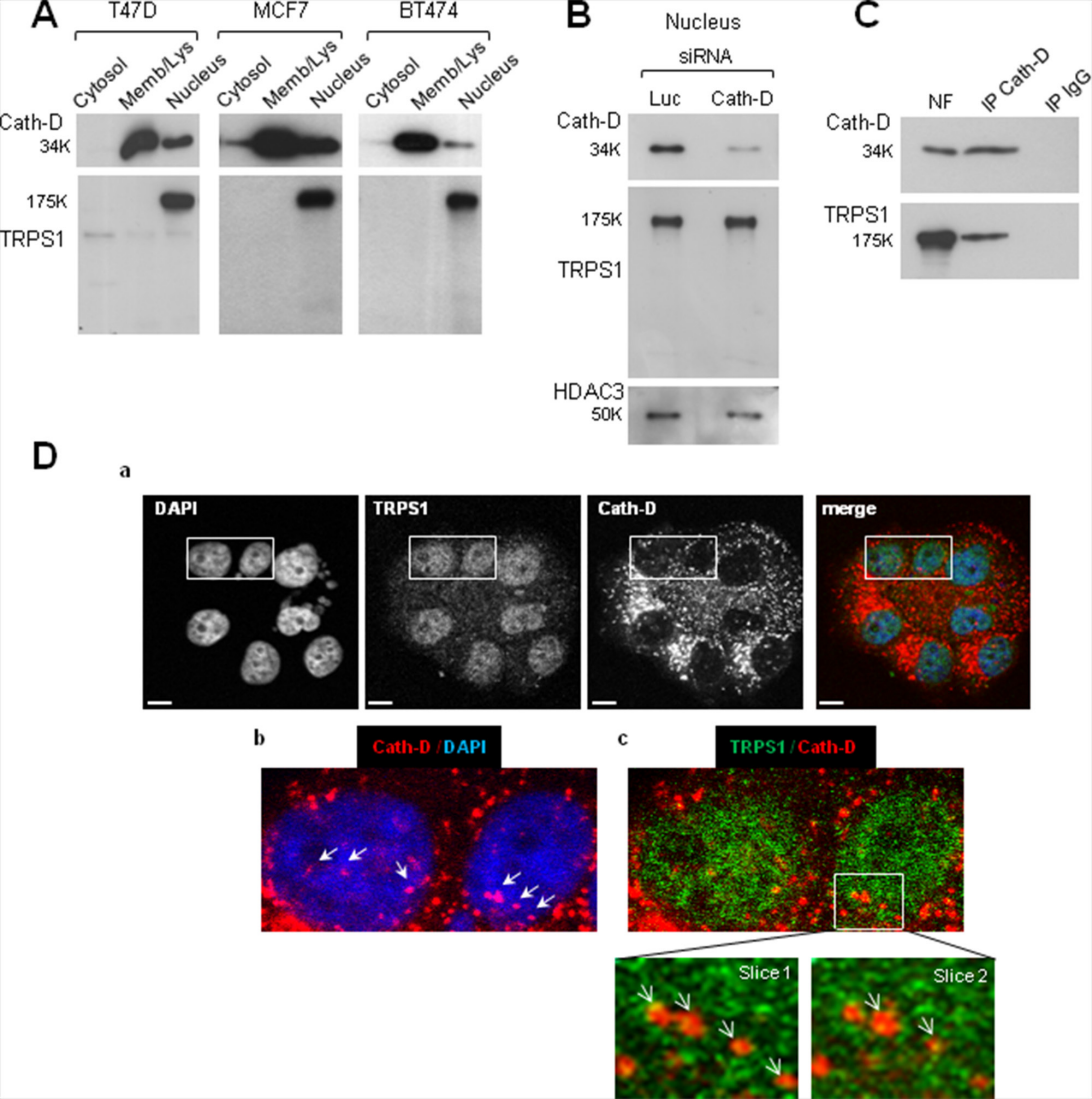
Nuclear interaction and co-localization of endogenous fully-mature Cath-D and full-length TRPS1 in ER^+^ BCC **A.** Nuclear Cath-D does not influence TRPS1 proteolysis. Cytosolic, membrane and nuclear fractions of T47D, MCF7 and BT474 cells (10 μg) were resolved by SDS-PAGE. TRPS1 and Cath-D expression in the different fractions were analyzed by immunoblotting. **B.** Nuclear Cath-D does not affect TRPS1 turnover. T47D cells were transfected with Cath-D siRNA2–3 (10 μg each) or control Luc siRNA (20 μg) for 48 h. Cath-D, TRPS1 and HDAC3 expression in the nuclear fraction was then assessed by WB. HDAC3, control for nuclear fraction. **C.** Endogenous TRPS1 and Cath-D are co-immunoprecipitated from nuclear fractions. Aliquots of T47D nuclear fraction (100 μg) were immunoprecipitated with the anti-Cath-D antibody M1G8 (IP Cath-D) or control IgG1 (IP IgG). Cath-D (top) and TRPS1 (bottom) were detected by WB. NF, nuclear fraction (10 μg). **D.** Co-localization of endogenous Cath-D and TRPS1 in the nucleus. Permeabilized T47D cells were double stained with the anti-Cath-D monoclonal antibody M1G8 (red) and an anti-TRPS1 polyclonal antibody (green). DNA was stained with 0.5 μg/ml DAPI (blue). Panel a: DAPI, Cath-D, TRPS1 and merge immunostaining analyzed by confocal microscopy (Z projections (max intensity) of 4 × 0.23 μm slices). Panel b: Cath-D immunostaining and Panel c: double immunostaining patterns. Higher magnifications (slices 1 and 2: 0.23 μm) are shown in the boxed regions. Arrows indicate Cath-D and TRPS1 nuclear co-localization. Scale bar: 10 μm.

We detected only intact full-length 175-kDa TRPS1 in the nucleus of ER^+^ BCC (Fig. 4A) and Cath-D silencing in T47D cells did not modify TRPS1 level (Fig. 4B). These findings suggest that TRPS1 binding to nuclear mature 34-kDa Cath-D does not induce limited or complete proteolysis of TRPS1. Immunoprecipitation of endogenous Cath-D in T47D nuclear extracts demonstrated that TRPS1 was immunoprecipitated together with mature 34-kDa Cath-D (Fig. 4C). Confocal immunocytochemistry and double staining with monoclonal anti-Cath-D, and polyclonal anti-TRPS1 antibodies (Fig. 4D) showed that Cath-D (in red; panel b) partially co-localized with TRPS1 (in green; panel c, see slices 1 and 2) in punctuate spots within the nucleus of T47D cells. Thus, fully-mature Cath-D and full-length TRPS1 interact and co-localize in the nucleus of ER^+^ BCC.

### BAT3 promotes Cath-D accumulation in the nucleus of ER^+^ BCC

**Figure 5:**
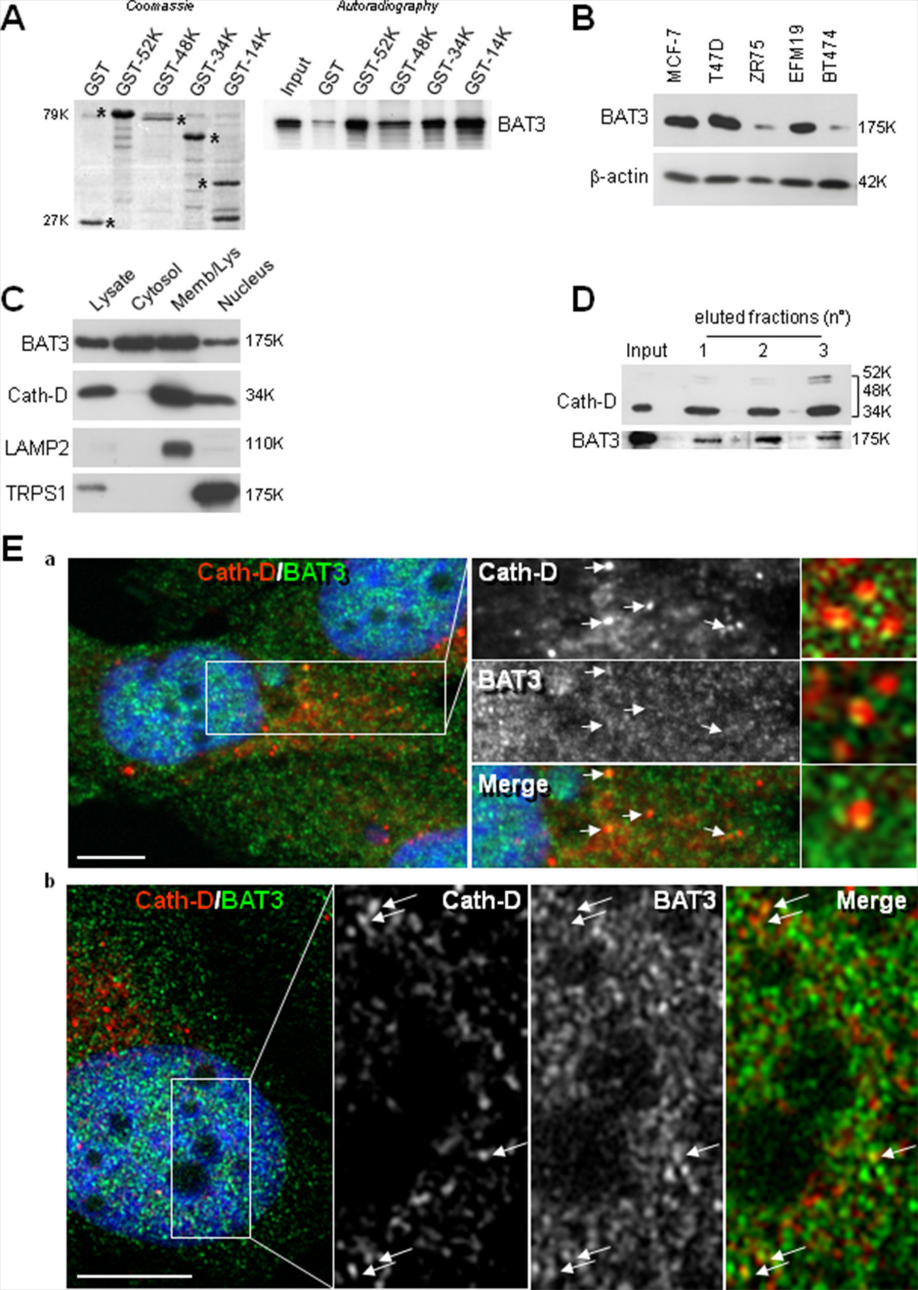
Binding of nuclear Cath-D to BAT3 **A.** Direct binding of BAT3 to Cath-D-GST. Radio-labeled full-length BAT3 was incubated with beads bearing GST-52K, -48K, -34K, -14K Cath-D, or GST. GST proteins stained with Coomassie blue are shown in the left panel. Bound BAT3 was detected by autoradiography (right panel). **B.** BAT3 expression in ER^+^ BCC lines. BAT3 expression was assessed in whole cell extracts (10 μg) by WB. β-actin: loading control. **C.** BAT3 subcellular localization. BAT3, Cath-D, LAMP2 (lysosomal marker) and TRPS1 (nuclear marker) expression were analyzed in the cytoplasmic, membrane and nuclear fractions (10 μg) of T47D cells by WB. **D.** Endogenous BAT3 is purified with Cath-D. T47D whole cell lysates were loaded on an anti-Cath-D M1G8 affinity column. Cath-D (top) and BAT3 (bottom) were detected in the unfractionated (input) and the three eluted fractions by WB. **E.** Co-localization of endogenous Cath-D and BAT3 in the nucleus. Permeabilized T47D cells were double stained with the anti-Cath-D monoclonal antibody M1G8 (red) and an anti-BAT3 polyclonal antibody (green). DNA was stained with 0.5 μg/ml DAPI (blue). Panel a: DAPI, Cath-D, BAT3 and merge immunostaining analyzed with a microscope equipped with Apotome to eliminate out-of-focus fluorescence (slices of ∼0.3 μm). Co-localization in lysosomes in the middle panels is indicated by arrows. The right panels shows details of vesicles with BAT3 spots located at the margins of lysosomes. Panel b: double immunostaining pattern in the nucleus showing Cath-D co-localization with BAT3 dots (arrows). Scale bar: 10 μm.

**Figure 6:**
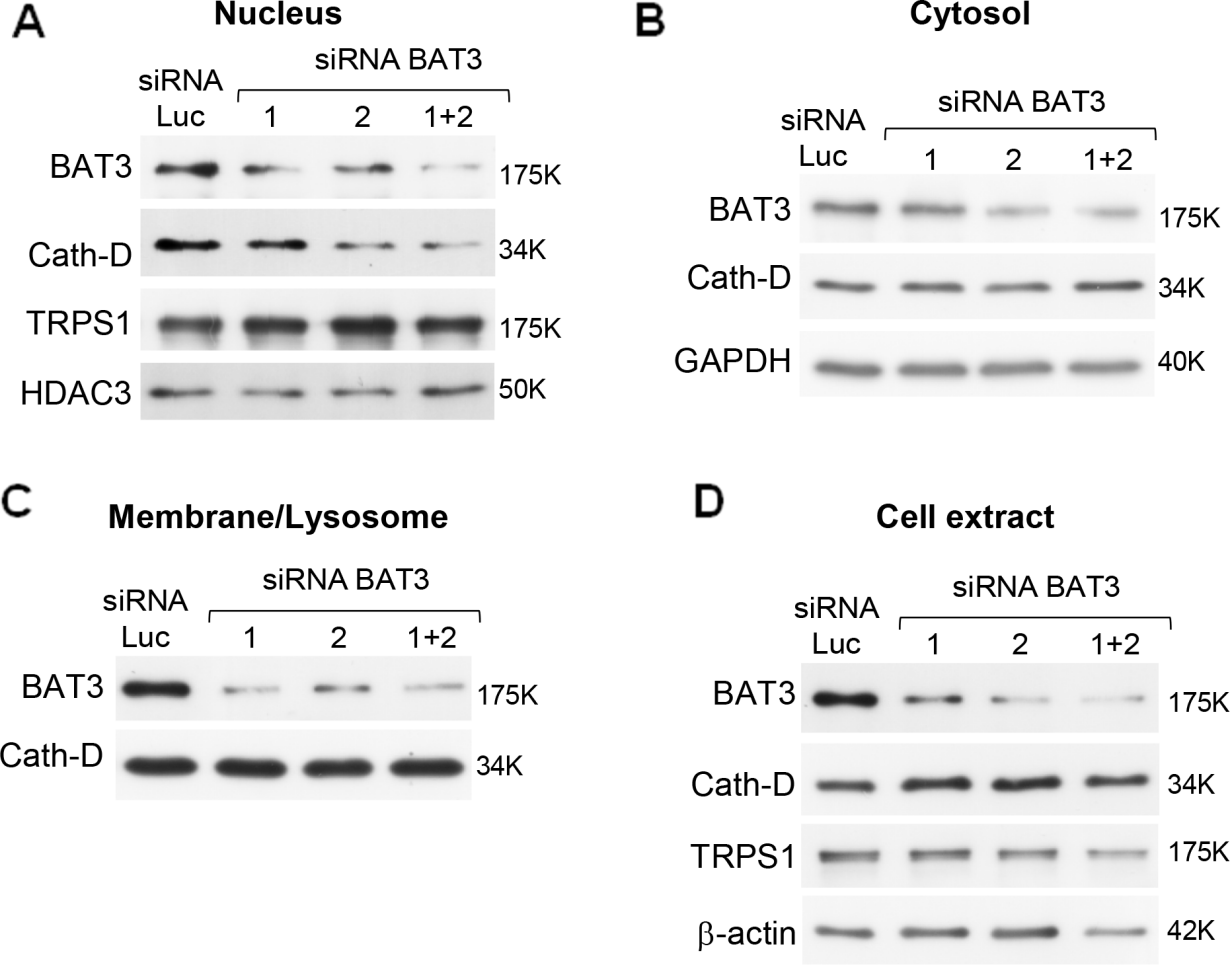
Regulation of nuclear Cath-D accumulation by its molecular partner BAT3 Nuclear (panel A), cytoplasmic (panel B), membrane (panel C) fractions, and whole cell extracts (panel D) (10 μg) from T47D cells transfected with Luc or BAT3 siRNA1 (20 μg), siRNA2 (20 μg), or siRNA(1+2) (10 μg each) were prepared 48 h post-transfection and BAT3, Cath-D, TRPS1, GAPDH, HDAC3 and β-actin expression analyzed by WB. GAPDH and HDAC3, loading markers for cytoplasmic and nuclear fractions. β-actin: whole cell extract loading control.

As Cath-D does not have a nuclear localization signal (NLS), we investigated whether mature Cath-D reaches the nucleus by binding to TRPS1. TRPS1 silencing did not prevent Cath-D accumulation in the nucleus of T47D cells (Fig. S2A). However, our yeast two-hybrid screen also identified BAT3 as a Cath-D partner (the clone was 100% identical to residues 376–659 in the proline-rich region of BAT3). BAT3 is a nucleo-cytoplasmic shuttling protein that contains an N-terminal ubiquitin homology region, a zinc finger-like domain, a nuclear export signal (NES), an NLS and, in its C-terminal part, a BAG domain that binds to the ATPase domain of HSC70/HSP70. We and others have shown that BAT3 targets the acetyltransferase p300 [28] and p21 [34] to the nucleus. We therefore investigated whether BAT3 binds to Cath-D by GST pull-down assay. All GST-Cath-D isoforms bound to BAT3, indicating that the interaction interface involves the whole Cath-D protein (Fig. 5A). Moreover, BAT3 was abundantly expressed in the tested ER^+^ BCC lines (Fig. 5B) and was detected in the membrane, cytoplasmic and nuclear fractions of T47D cells (Fig. 5C). Binding of endogenous Cath-D to endogenous BAT3 in T47D cells was confirmed by immunoaffinity purification with the M1G8 anti-Cath-D antibody (Fig. 5D). Confocal immunocytochemistry in T47D cells (Fig. 5E) showed that Cath-D (in red; panels a) partially co-localized with BAT3 (in green; panels a) in vesicle-like structures surrounding the nucleus (panels a; arrows). At higher magnification, BAT3 dots were frequently seen at the margins of lysosomes (panels a, right) and some punctuate Cath-D/BAT3 spots within the nucleus (arrows in panels b). Finally, BAT3 silencing in T47D cells with different BAT3 siRNAs (siRNA 1, 2 or 1+2) reduced the nuclear accumulation of Cath-D, but not of TRPS1 (Fig. 6A). Concomitantly, Cath-D expression was slightly increased in the cytosol of T47D cells silenced with the BAT3 siRNAs 1+2 (Fig. 6B). Cath-D lysosomal expression was unaffected by BAT3 silencing (Fig. 6C). Similarly, BAT3 silencing in T47D cells did not modify Cath-D or TRPS1 expression in whole cell extracts (Fig. 6D). These results strongly suggest that Cath-D nuclear accumulation is controlled by the chaperone protein BAT3. The finding that mature Cath-D in T47D nuclei is glycosylated indicates that it had passed through the endoplasmic reticulum (ER) (Fig. S2B).

### Transcriptional repression by Cath-D

**Figure 7:**
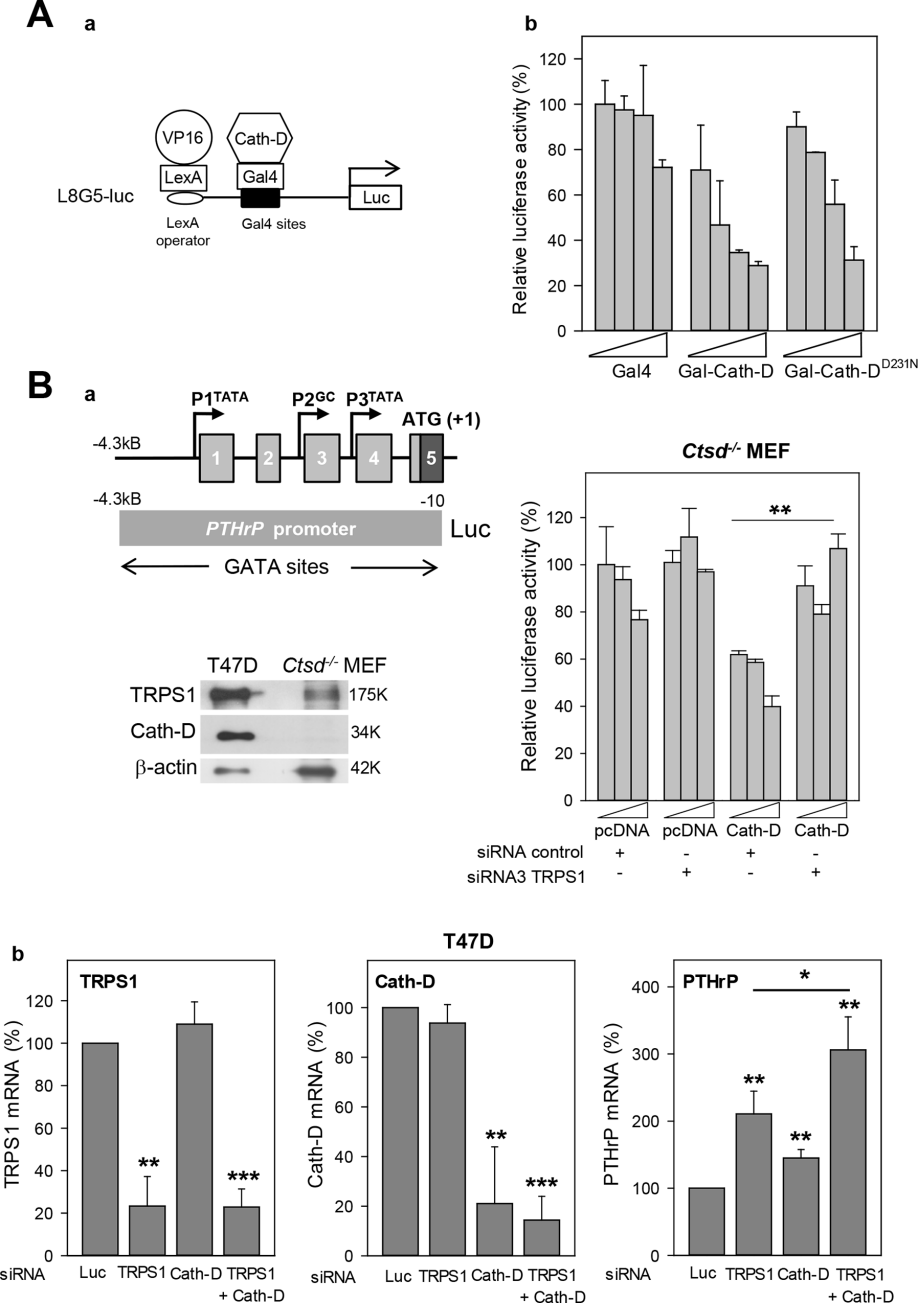
Transcription repression by TRPS1 and Cath-D **A.** Transcription repression by Cath-D Panel a: Diagram of the L8G5-Luc reporter gene containing the LexA operator sequence and Gal4 binding sites. LexA-VP16 is a transcription transactivator. Gal4 fusion proteins (Gal-Cath-D, Gal-^D231N^Cath-D) bind to the Gal4 sites to modulate LexA-VP16-induced transcription. Panel b: T47D cells were transfected with pRL-CMV (Renilla) (40 ng), the L8G5-Luc reporter gene (160 ng), the LexA-VP16 expression plasmid (80 ng) and increasing concentrations (100 to 800 ng) of the plasmids encoding the indicated Gal4 fusion proteins. Data are the percentage of the standardized luciferase activity obtained with Gal4 (100 ng) and are the mean ± SD of triplicate transfections. Similar results were obtained in another independent experiment. **B.** Regulation of the *PTHrP* promoter activity by TRPS1 and Cath-D. Panel a: The upper left image shows a diagram of the 5′-flanking 4.3 kB region of the human *PTHrP* gene and the *PTHrP* promoter-luciferase construct. Exons are indicated by boxes and promoters by arrows. Lower left image: Expression of TRPS1 and Cath-D was analyzed in whole cell extracts from *Ctsd−* MEFs and T47D cells by western blot analysis. β-actin, loading control. Histogram on the right: *Ctsd*^−/−^ MEFs were co-transfected with pRL-CMV (Renilla) (40 ng), the pGL2-PTHrP promoter plasmid or pGL2 empty vector (250 ng), and increasing concentrations of Cath-D expression plasmid or pcDNA3 empty vector (200, 300 and 400 ng) in the presence of a non-specific control siRNA (5′AGGUAGUGUAAUCGCCUUGdTdT 3′) or TRPS1 siRNA3 (125 ng). No luciferase activity was detected in cells with the pGL2 empty vector. **p < 0.01, Student’s *t*-test. Similar results were obtained in another independent experiment. Panel b: T47D cells were transfected in triplicate with Luc siRNA (20 μg), anti-TRPS1 or anti-Cath-D siRNA1–3 (20 μg), or with both TRPS1 and Cath-D siRNA2–3 (5 μg of each, total 20 μg). *CTSD*, *TRPS1* and *PTHrP* mRNA levels were determined by RT-qPCR 48 h later. Mean ± SD of three independent transfections. **p < 0.01; ***p < 0.0005; Student’s *t*-test.

As Cath-D binds to TRPS1, we investigated whether it can regulate gene transcription using a heterologous transcription assay. The reporter system used contained eight copies of the LexA binding site adjacent to five copies of the GAL4 binding site [35], cloned upstream of the luciferase gene (Fig. 7A, panel a). In the presence of the LexA-VP16 fusion co-activator (VP16) and the GAL4 DNA-binding domain (Gal4), this reporter was strongly activated in T47D cells (data not shown). Full-length wild type and the D231N Cath-D mutant, which is proteolytically inactive, were fused to the GAL4 DNA-binding domain (Gal-Cath-D, Gal-Cath-D^D231N^). Co-expression of LexA-VP16 and Gal-Cath-D inhibited luciferase activity in a dose-dependent manner in T47D cells (Fig. 7A, panel b). Similar results were obtained with ^D231N^Cath-D, indicating that Cath-D enzymatic activity is not required for transcriptional inhibition (Fig. 7A, panel b). We thus hypothesized that the Cath-D/TRPS1 molecular interplay regulates TRPS1 transcriptional repressor activity. In order to explore this possibility, we next studied the impact of Cath-D on the transcription of endogenous TRPS1 target genes.

### Cath-D enhances the transcriptional repressor function of TRPS1

To determine whether Cath-D binding to TRPS1 affects TRPS1 transcriptional repressor activity, we first assessed the effect of TRPS1 silencing (Fig. S3) on the transcription of endogenous TRPS1-target genes, such as *parathyroid hormone-related protein* (*PTHrP*) [21], *STAT3* [22], *osteocalcin* [36] and *ZEB2*, which induces EMT in BCC [24]. TRPS1 silencing in T47D cells significantly (2.1-fold, *p* < 0.005) increased only *PTHrP* mRNA expression, but did not affect *ZEB2, STAT3* and *osteocalcin* expression (Fig. S4). Therefore, the 4.3 kB *PTHrP* promoter region, which contains multiple GATA, Ets-1 and CBP binding sites, was fused to a luciferase reporter gene (Fig. 7B, panels a, left top image) to determine whether Cath-D affects TRPS1 transcriptional repressor activity. Luciferase activity indicated that the *PTHrP* promoter was active in *Ctsd*^−^ MEFs, but not in wild type T47D cells (not shown). However, Cath-D re-expression in *Ctsd*^−^ MEFs repressed the transcriptional activity of the 4.3 kB *PTHrP* promoter in a dose-dependent manner and this effect was counteracted by TRPS1 silencing (Fig. 7B, panel a, histogram on the right), suggesting that Cath-D cooperates with TRPS1 in inhibiting *PTHrP* transcription. Indeed, TRPS1 and Cath-D co-silencing in T47D cells (reduction of their expression by 80%) further increased *PTHrP* mRNA expression compared to silencing of TRPS1 alone, indicating that Cath-D potentiates (1.5-fold, *p* < 0.05) *PTHrP* transcriptional repression by TRPS1 (Fig. 7B, panels b).

**Figure 8:**
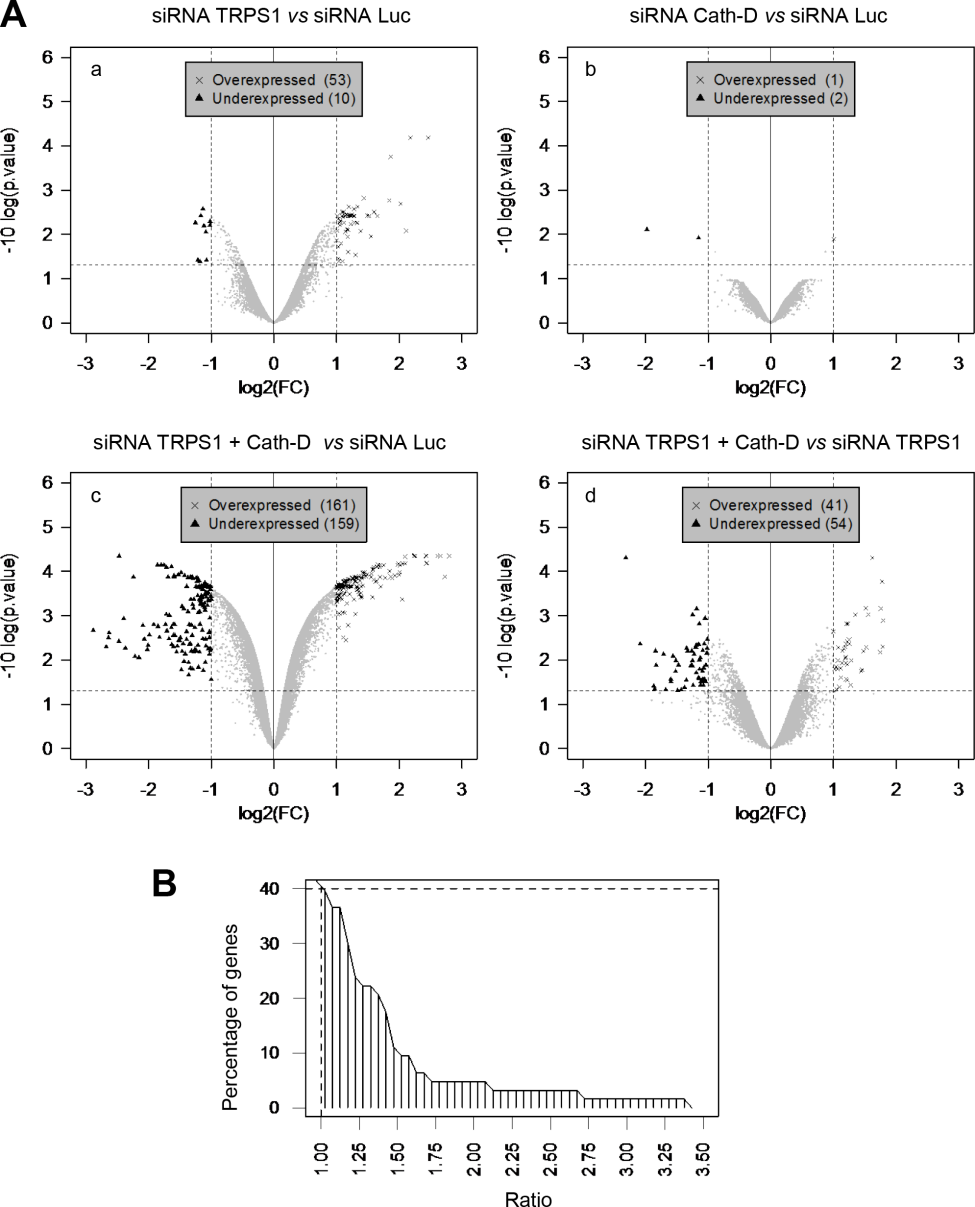
Cath-D influences TRPS1 transcription repression function **A.** Transcriptome analysis of TRPS1-silenced and Cath-D-silenced cells. T47D cells were transfected in triplicate with control Luc siRNA (20 μg), TRPS1 or Cath-D siRNA1–3 (20 μg), or with both TRPS1 and Cath-D siRNA2–3 (5 μg of each, total 20 μg). RNAs were extracted 48 h post-transfection and analyzed by microarray hybridization. Panel a: TRPS1-silenced cells *versus* control. Panel b: Cath-D-silenced cells *versus* control. Panel c: TRPS1/Cath-D doubly-silenced cells *versus* control. Panel d: TRPS1/Cath-D doubly-silenced cells *versus* TRPS1-silenced cells. The significant differences in the mRNA expression of a given gene were determined by dividing the log2 of TRPS1, Cath-D or TRPS1/Cath-D silenced sample signals by the Luc control signal, *i.e*. log(FC); these data were analyzed using a modified Student’s *t*-test followed by correction for multiple testing. Over- and under-expressed genes, relative to control, were identified using a threshold for the log(FC) (X-axis) (between 1 or −1), corresponding to fold-changes of 2 and −2, and a threshold for the adjusted *p* value of 0.05 (Y-axis). **B.** Cumulative frequency distribution of the difference (fold-change) between TRPS1/Cath-D co-silencing and TRPS1 silencing alone. T47D cells were transfected with Luc siRNA (20 μg), or TRPS1 + Cath-D siRNA2–3 (5 μg of each, total 20 μg) in triplicate transfections. Microarray analysis showed the impact of double silencing on the 53 mRNAs that were significantly up-regulated following silencing of TRPS1 alone (Table S1). Ratio (x-axis), ratio of fold-changes for the 53 mRNAs after TRPS1 and Cath-D co-silencing divided by the fold-changes after TRPS1 silencing.

Finally, to determine whether Cath-D promotion of TRPS1 transcriptional repression was not confined to *PTHrP*, the transcriptomes of T47D cells transfected with siRNAs against Cath-D or/and against TRPS1, or with the control Luc siRNA were analyzed by microarray analysis. Compared to control (Luc siRNA), TRPS1 silencing led to a significant (2-fold) increase in 53 mRNAs and decrease in ten others (Fig. 8A, panel a; Tables S1 and S2). Cath-D silencing significantly up-regulated (2-fold) only *ARL6IP5* (ADP-ribosylation-like factor 6 interacting protein 5) (Fig. 8A, panel b; Table S3). TRPS1 and Cath-D co-silencing led to significant up-regulation of 161 genes and down-regulation of 159 (Fig. 8A, panel c; Table S3). Comparison of the genes affected by TRPS1/Cath-D co-silencing with those affected by TRPS1 silencing showed that 41 genes were significantly up-regulated, while 54 genes were down-regulated in co-silenced cells (*p* < 0.00001) (Fig. 8A, panel d). Specifically, TRPS1/Cath-D co-silencing potentiated by 1.4-fold the expression of 10 of the 53 mRNAs up-regulated by TRPS1 silencing alone (Fig. 8B; Table S1). This suggests that Cath-D enhances TRPS1 transcriptional inhibition activity. RT-qPCR confirmed that *RBP1* (retinol-binding protein I) and *MAOA* (monoamine oxidase A), two of the most up-regulated genes in TRPS1-silenced T47D cells compared to control, were significantly more over-expressed in TRPS1/Cath-D co-silenced than in TRPS1-silenced T47D cells (Fig. S5).

### Impact of Cath-D/TRPS1 silencing on cell cycle progression and transformation

**Figure 9:**
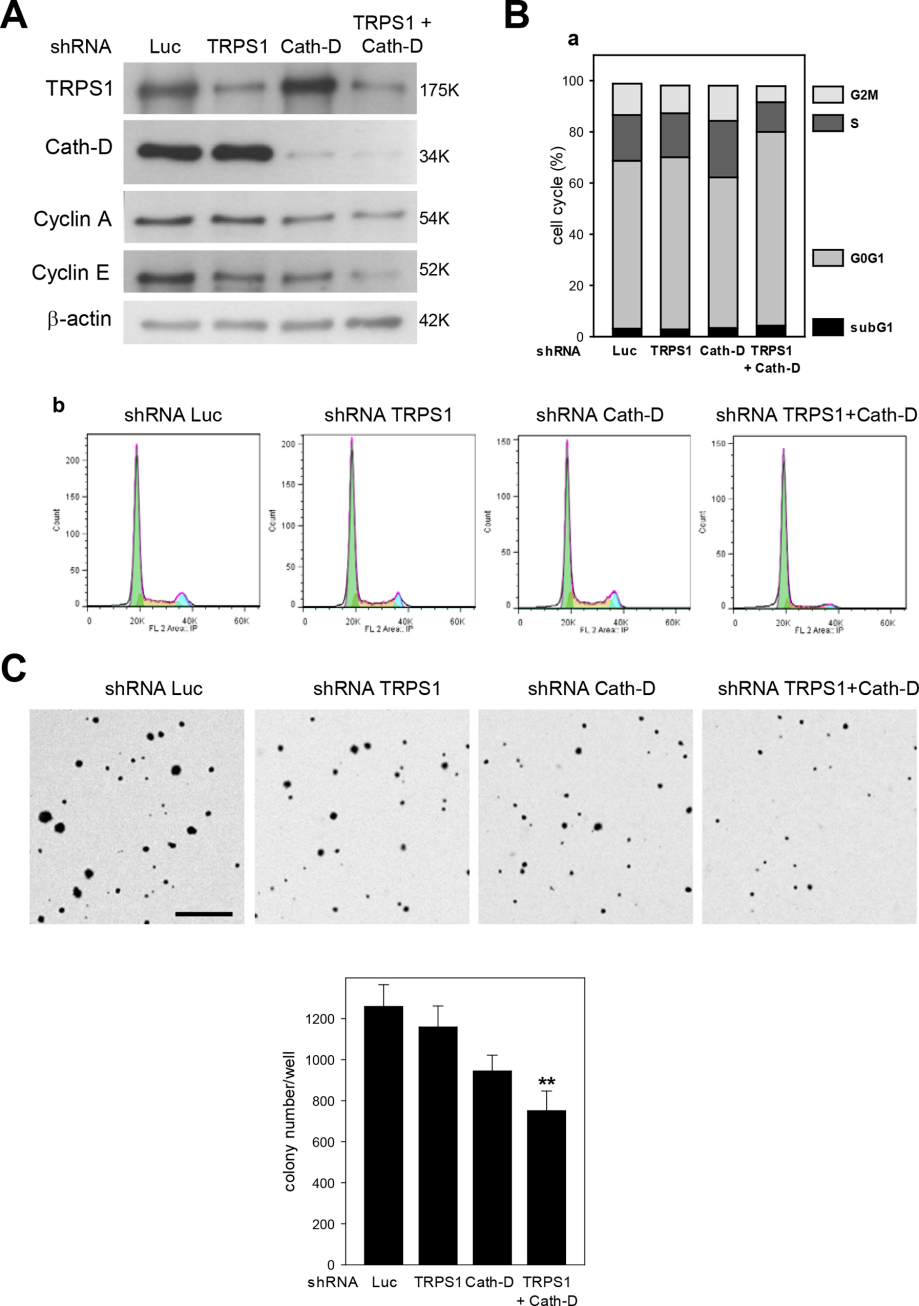
Cath-D/TRPS1 dual silencing inhibits cell cycle progression and cell transformation **A.** Expression of cyclin A and E. T47D cells were transfected with Luc, TRPS1, Cath-D or TRPS1+Cath-D shRNAs. Five days post-transfection, TRPS1, Cath-D, cyclin A and E expression were analyzed in whole cell extracts (10 μg) by western blotting. β-actin: loading control. Similar results were obtained in three independent experiments. **B.** Cell cycle analysis. Cell cycle progression in T47D cells was analyzed by flow cytometry five days after transfection with the indicated shRNAs. Cell cycle analysis (panels b) and quantification of the subG1, G0G1, S and G2M fractions (panel a). Similar results were obtained in three independent experiments. **C.** Soft agar colony formation. Three days post-transfection, T47D cells were embedded in soft agar and grown for 8 days. The resulting colonies were stained with p-iodonitrotetrazolium violet and phase-contrast photomicrographs taken. Colonies/well in 6-well plates were counted using Image J (Panel b). Data are the mean ± SD relative to Luc shRNA (*n* = 6). **p < 0.01, Student’s *t*-test. One of two representative experiments is shown. Bar = 500 μm.

Our microarray analysis indicated that TRPS1/Cath-D co-silencing variably affected (up or down-regulation) the expression of 26 cell cycle genes compared to TRPS1 silencing alone (five genes) (*p* < 0.00001) (Table S4) and 79 proliferation genes (only ten in TRPS1-silenced cells *p* < 0.00001) (Table S5). In addition, TRPS1/Cath-D co-silencing led to significant changes in the expression of 15 transformation genes (*p* < 0.00001), whereas TRPS1 silencing affected only three transformation genes (Table S6). Analysis of cell cycle progression by flow cytometry in unsynchronized T47D cells showed that TRPS1 and Cath-D co-silencing (Fig. 9A) inhibited cell cycle progression with cell accumulation in the G0/G1 phase and reduced number of cells in the S and G2/M phases (*p* < 0.025) (Fig. 9B, panels a and b). No significant effect was observed on the subG1 phase, indicating that apoptosis was not affected by TRPS1 and Cath-D co-silencing. Inhibition of cell cycle progression in co-silenced cells was also associated with decreased levels of cyclin E, a marker of the S phase, and cyclin A, a marker of the S and G2/M phases (Fig. 9A). Moreover, investigation of the effect of TRPS1 and/or CathD silencing on soft agar colony formation, a signature of cell transformation *in vitro*, showed that TRPS1/Cath-D co-silencing in T47D cells resulted in fewer and smaller colonies (40% decrease) than in control (Luc shRNA) cells (Fig. 9C). Of note, in the polyoma middle-T antigen transgenic model of luminal ER^+^ breast cancer, TRPS1 mRNA expression was significantly up-regulated (8-fold) in the earliest hyperplastic lesions in 5-week/old transgenic mice compared to mammary glands from wild type mice (Fig. S6). Our findings therefore strongly suggest that the TRPS1/Cath-D interplay is implicated in the progression of luminal ER^+^ breast cancer.

## DISCUSSION

Here, we report that Cath-D accumulates in the nucleus of ER^+^ BCC and the identification of new Cath-D nuclear partners (*i.e*., TRPS1 and BAT3). We found that Cath-D interacts with the GATA family member TRPS1 in the nucleus of ER^+^ BCC and enhances TRPS1 transcription repressor activity, independently of its proteolytic function. Both the 34-kDa heavy and 14-kDa light chains of Cath-D bind to a 200 aa TRPS1 region located between the GATA DNA-binding zinc finger and the C-terminal IKAROS-like double zinc fingers. This TRPS1 region also interacts with the RING finger protein RNF4 that counteracts TRPS1-mediated repression [37].

In the nucleus, Cath-D association with chromatin could be due to translation initiation at alternative, downstream AUG sites and synthesis of a protease that lacks the N-terminal hydrophobic signal peptide. N-terminally truncated Cath-D isoform could escape transit through the ER, leading to nuclear accumulation of unglycosylated Cath-D. However, Cath-D detected in ER^+^ BCC nuclei was the fully mature glycosylated enzyme. Moreover, neither mouse nor human Cath-D has an out-of-frame start codon and such codons hinder the biosynthesis of truncated cathepsin L [38]. This strongly supports the hypothesis that nuclear Cath-D is not an N-terminally truncated variant, but the fully mature form, normally found in lysosomes. The nuclear pool of Cath-D could be due to low-level lysosomal permeabilization or retrograde protein trafficking of the fully mature enzyme [39].

As Cath-D does not have a NLS, we hypothesized that its nuclear targeting could be mediated by binding to TRPS1, which has an NLS [40]. However, TRPS1 silencing did not change the amount of nuclear Cath-D. On the other hand, in ER^+^ BCC, Cath-D also binds to BAT3, a nucleo-cytoplasmic shuttling protein and BAT3 silencing inhibits Cath-D nuclear accumulation. There is evidence that cytosolic BAT3 captures soluble or membrane-anchored proteins and transfers them to organelles, such as the ER, or the nucleus *via* formation of multiple protein complexes involving the membrane interface [29, 34, 41]. Based on the finding that Cath-D/BAT3 can co-localize at the surface of lysosomes, we suggest that BAT3 captures the excess Cath-D that escapes from lysosomes and helps transporting the protease to the nucleus of ER^+^ BCC. As BAT3 can trigger p21 nuclear targeting during the cell cycle [34], future studies are needed to evaluate the regulation of Cath-D nuclear accumulation in proliferating and non-proliferating ER^+^ BCC.

We then show that Cath-D acts as a transcription co-repressor. This new intracrine mechanism is independent of its proteolytic function because the D231N Cath-D mutant, which is proteolytically inactive, retains this inhibitory activity in the (LexA/VP16) luciferase reporter promoter assay. Similarly, the D231N Cath-D mutant is still mitogenic in cancer cells [10, 12, 42], indicating that Cath-D has additional actions independently of its catalytic activity. Moreover, Cath-D enhances TRPS1-mediated gene transcription repression in ER^+^ BCC. Importantly, fully mature Cath-D binds to full-length TRPS1 in ER^+^ BCC nuclei and Cath-D silencing does not alter TRPS1 level, suggesting that Cath-D effect on transcription is mediated through a non-proteolytic chaperoning mechanism. Conversely, nuclear cysteine Cath-L modulates transcription through limited proteolysis of the CDP/Cux transcription factor and of histone H3 [43, 44]. We also provide evidence that the co-repressive action of Cath-D and TRPS1 is quite widespread. Indeed, the expression of 40% of the 53 TRPS1-repressed genes we identified by microarray analysis was more strongly up-regulated (ratio ≥ 1) in Cath-D/TRPS1 co-silenced cells than in TRPS1 silenced cells.

The results of the transcriptome analysis in TRPS1 and/or Cath-D silenced cells suggest that the TRPS1/Cath-D interplay could have dual (oncogenic or anti-oncogenic) effects in ER^+^ BCC. Indeed, TRPS1 and Cath-D co-repress transcription of *secreted protein rich in cysteine gene* (*SPARC)*, a tumor suppressor in MDA-MB-231 ER^−^ BCC [45] and promote *c-MYC* and *TGFB3* expression. The *MYC* oncogene is co-amplified with TRPS1 in breast carcinomas with increased proliferation rate [46]. The pleiotropic cytokine TGFβ3 promotes ER^+^ BCC invasive potential [47]. However, Cath-D and TRPS1 also co-inhibit transcription of genes implicated in tumor progression, as shown for *PTHrP* that encodes a secreted factor driving bone metastasis formation in breast cancer. PTHrP is weakly expressed in ER^+^ BCC and its overexpression in ER^+^ MCF7 leads to bone metastasis formation [48]. Triple-negative (ER^−^, PR^−^, HER2^−^) metastatic BCC require PTHrP for their proliferation and survival [49]. TRPS1 is strongly expressed in ER^+^ BCC, such as T47D cells, while its level is lower in triple-negative MDA-MB-231 BCC [23]. Thus, nuclear Cath-D and TRPS1 may help limiting the tumorigenic and metastatic capacity of T47D cells by repressing *PTHrP* gene expression. We also found that the Cath-D/TRPS1 pair reduces the transcription of oncogenic elements, including the KIT ligand (*KITLG*) and *c-MYB*. Thus, our finding that TRPS1/Cath-D co-regulate a subset of genes encoding oncogenic or anti-oncogenic effectors, which are implicated in cell proliferation, differentiation and transformation, supports the pleiotropic and opposing biological functions associated with TRPS1 [19–22, 50].

While TRPS1 and Cath-D seem to have a dual effect on several genes involved in carcinogenesis, TRPS1/Cath-D co-silencing in ER^+^ T47D cells inhibits cell cycle progression and anchorage-independent growth. Many reports indicated that Cath-D is mitogenic in BCC [5–8, 10–12]. More recently, high Cath-D levels were associated with poor prognosis in patients with ER^+^ breast cancer [51, 52]. Moreover, TRPS1 is overexpressed in breast cancer [23] and its expression is linked to the ER^+^, GATA3 and HER2 status [53–55] and to the EMT-negative phenotype, as shown here for TRPS1 and Cath-D [24]. TRPS1 was proposed to have a prognostic value in early stage breast cancer [56] and to promote tumor progression [57]. In addition, TRPS1 stimulates cell proliferation [25] and angiogenesis [58] in breast cancer. Altogether, these data indicate that TRPS1 and Cath-D are implicated in luminal breast tumor oncogenesis by promoting cell cycle progression and maintaining the transformed phenotype in ER^+^ BCC.

In summary, our study provides evidence that nuclear Cath-D is a TRPS1 molecular partner that enhances TRPS1 repressor activity in luminal ER^+^ BCC. Our studies provide a template for the preclinical evaluation of critical Cath-D/TRPS1 target genes involved in the progression of specific breast cancer subtypes.

## MATERIALS AND METHODS

### Materials

The entire coding sequence of human TRPS1 was cloned in the pcDNA4 plasmid and the TRPS1 fragments F1, F5, F6, F9 and F10 were cloned in the pGADT7 plasmid [37]. Full-length BAT3 cDNA cloned in the pcDNA3-HA vector was a generous gift from F. Desmots-Loyer (CHU Hôpital Pontchaillou, Laboratoire d’Hématologie, Rennes, France). The pGEX-4T-1-Cath-D constructs were obtained by inserting PCR-amplified cDNAs encoding the human 52-, 48-, 34- or 14-kDa Cath-D chains in pGEX-4T-1 that was previously digested with *Eco*RI [14]. The PTHrP-luciferase reporter plasmid containing the 4.3 kb BamHI-HindIII *PTHrP* promoter region cloned in the pGL-2 vector upstream of the luciferase gene (Promega, USA) was kindly provided by Z. Bouizar (INSERM U349, Paris, France). The L8G5-Luc and LexA-VP16 constructs were kindly donated by V. Cavailles (INSERM U896, IRCM Montpellier, France). Gal4-Cath-D and Gal4-^D231N^Cath-D were obtained by inserting the 1.2 kb Cath-D or ^D231N^Cath-D cDNA into the *Eco*RI site of the pM vector (Clontech Laboratories). BCC and HMFs were cultured in DMEM with 10% fetal calf serum (FCS, GibcoBRL, Life Technologies, Carlsbad, CA, USA). HMFs, kindly provided by J. Piette (IGM, Montpellier, France), were obtained from reduction mammoplasty tissue from a patient without cancer. HMT-3522-S1 cells, kindly provided by P. Briand (Department of Gynecology and Obstetrics, Rigshospitalet, Copenhagen, Denmark), were cultured in H14 chemically defined growth medium. Snail-MCF7 and Snail6SA-MCF7 cells were kindly provided by Pr M.C. Hung [33]. Purified goat anti-human TRPS1 antibody and purified normal goat IgG were purchased from R&D Systems (Minneapolis, MN, USA). The rabbit polyclonal anti-human BAT3 antibody used for western blotting was a generous gift from F. Desmots-Loyer (CHU Hôpital Pontchaillou, Laboratoire d’Hématologie, Rennes, France). The anti-human Cath-D monoclonal antibody (BD Biosciences, San Jose, CA, USA) used for immuno-blotting and the anti-human Cath-D monoclonal IgG1 antibody M1G8 used for immunoprecipitation recognize the 52-, 48- and 34-kDa forms of Cath-D. Rabbit polyclonal anti-human LAMP2 and chicken polyclonal anti-BAT3 antibodies were purchased from Abcam (Cambridge, UK). The control IgG1 anti-MOPC-21 monoclonal antibody and anti-β actin polyclonal antibody were purchased from Sigma-Aldrich (St Louis, MO, USA). The mouse monoclonal anti-GAPDH (6C5), rabbit polyclonal anti-human HDAC3 (H-99), rabbit polyclonal anti-cyclin A (H-432) and the rabbit polyclonal anti-histone H3 antibody were from Calbiochem (Merck, Germany). The monoclonal mouse anti-human E-cadherin antibody was from BD Transduction Laboratories (San Jose, CA, USA), the monoclonal mouse anti-vimentin antibody (Clone V9) from DakoCytomation (Glostrup Denmark), the mouse monoclonal anti-α-tubulin (clone DM1A; epitope 426–450) from Lab Vision Corporation (Thermo Fisher Scientific Inc., Rockford, USA) and the mouse monoclonal anti-cyclin E (clone HE12) antibody from Millipore. The mouse monoclonal anti-ERK2 (D-2) antibody was purchased from Santa Cruz Biotechnology (Cruz, CA, USA). Endoglycosidase H was purchased from Roche Applied Science (Indianapolis, IN, USA).

### Breast tumors

This project was submitted to the Ethics Committees of the clinical centers taking part in the study and was approved by the National Institute of Cancer (INCa), following the recommendations of the French National Authority for Health (FNAH). Patient samples were processed according to the French Public Health Code (law n°2004–800, articles L. 1243–4 and R. 1243–61) and the biological resources center has been authorized (authorization number: AC-2008–700; Val d’Aurelle, ICM, Montpellier) to deliver human samples for scientific research. All patients were informed before surgery that their surgical specimens might be used for research purposes. They could refuse by completing an appropriate form; their tumor biopsies would then be destroyed. A total of 44 primary breast cancer samples were obtained from the Pathology Department. This study was reviewed and approved by the Montpellier Cancer Center - Val d’Aurelle Institutional Review Board and informed consent was obtained from all patients. Samples were systematically anonymized. RNAs were isolated from frozen tissues using the RNeasy Mini Kit (Qiagen S.A. France, Courtaboeuf, France) and their quality/quantity assessed on a Bioanalyser (Agilent, Santa Clara, CA, USA).

### Yeast two-hybrid screening

Yeast two-hybrid screening was performed by Hybrigenics Services, S.A.S., Paris, France (http://www.hybrigenics-services.com). Briefly, a fragment containing aa 65–412 of human Cath-D (GenBank accession number gi: 29677) fused in frame with the Gal4 DNA-binding domain was used to screen a randomly-primed human breast tumor epithelial cell cDNA library.

### GST pull-down assay

[^35^S]methionine-labeled full-length TRPS1 (or BAT3) and the TRPS1 fragments were obtained by transcription and translation using the TNT^T7^-coupled reticulocyte lysate system (Promega). The production of GST and GST-Cath-D fusion proteins in the *Escherichia coli B* strain BL21 was induced by incubation with 1 mM isopropyl-1-thio-β-D-galactopyranoside at 37°C for 3 h. The GST fusion proteins were purified on glutathione-Sepharose beads (Amersham Biosciences, USA). 20 μl aliquots of glutathione-Sepharose beads bearing immobilized GST fusion proteins were incubated overnight at 4°C with [^35^S]methionine-labeled proteins in 500 μl PDB buffer (20mM HEPES-KOH (pH 7.9), 10% glycerol, 100 mM KCl, 5 mM MgCl_2_, 0.2 mM EDTA, 1 mM DTT, 0.2 mM PMSF) containing 15 mg/ml BSA and 0.1% Tween-20. Proteins were eluted after four washes (500 μl each) of PDB buffer. Eluted proteins were resolved on 15% SDS-PAGE, stained with Coomassie blue, and bands detected on exposed X-ray films.

### siRNA and shRNA transfection

21-nucleotide siRNA duplexes against human Cath-D (target sequence AAGCUGGUGGACCAGAACAUC; siRNA1 [14]) were synthesized by Dharmacon RNA Technologies (Thermo Fisher Scientific Inc., Rockford, IL, USA), and the siRNA against firefly luciferase (Luc) (target sequence AACGUACGCGGAAUACUUCGA) by Qiagen Sciences (Maryland, USA). Human Cath-D siRNA2 (ID 105581) and siRNA3 (ID 4180) were purchased from Ambion (Austin, TX). Human TRPS1 siRNAs were purchased from Thermo Scientific Dharmacon (ON-TARGET *plus*: siRNA1 (J-009644–05), siRNA2 (J-009644–06), siRNA3 (J-009644–08)). T47D cells grown in 6-well plates were transiently transfected with Luc, Cath-D, or/and TRPS1 siRNAs using Lipofectamine (Invitrogen, Life Technologies, USA) by incubation at 37°C for 48 h. Cells were then lysed in 50 mM Hepes [pH 7.5], 150 mM NaCl, 10% glycerol, 1% Triton X-100, 1.5 mM MgCl_2_ 1 mM EGTA and a protease inhibitor cocktail. Total RNA was extracted from the lysates using the RNeasy Mini Kit (Qiagen Sciences, Maryland). T47D cells were also transfected with 1 μg Luc, Cath-D, and/or TRPS1 shRNA expression vectors (Invivogen, San Diego, CA, USA) using Nucleofector Technology (Amaxa biosystems, MD, USA). Sequences are shown in Table S7.

### Subcellular fractionation, immunoprecipitation, immunoblotting, immunopurification, and flow cytometry

Subcellular cytoplasmic, membrane and nuclear fractions of BCC were harvested using the Subcellular Protein Fractionation Kit (Thermo Scientific, USA). Cytoplasmic, membrane and nuclear extracts (10 μg) were separated by SDS-PAGE and immunoblotted with anti-Cath-D, anti-TRPS1, anti-LAMP2, anti-GAPDH and anti-HDAC3 antibodies using standard techniques. For co-immunoprecipitation experiments, nuclear extracts (100 μg) of T47D cells were incubated with 1 μg anti-Cath-D M1G8 monoclonal antibody, or 1 μg of anti-MOPC21 control IgG1 monoclonal antibody at 4°C overnight, and then with 50 μl 10% protein A-Sepharose at 4°C on a shaker for 2 h. Sepharose beads were washed thrice in 50 mM Hepes [pH 7.5], 150 mM NaCl, 10% glycerol, 1% Triton X-100, 1 mM EGTA and once with the same buffer containing 300 mM NaCl, boiled for 5 min in SDS sample buffer, and analyzed by SDS-PAGE. Samples were then immunoblotted with the anti-Cath-D monoclonal antibody or anti-TRPS1 polyclonal antibody. Cath-D was purified from cells lysed in lysis buffer (100 mM Tris/HCl [pH 8], 100 mM NaCl, 1% NP40 and 1 mM DTT) containing protease inhibitors. Lysates were purified through an agarose column coupled with 1 ml anti-Cath-D M1G8 antibody. The column was washed with phosphate buffer (0.5 M NaPO_4_, 150 mM NaCl, 0.01% Tween 80, 5 mM β-glycerophosphate) and proteins eluted in three 500 μl fractions with 20 mM lysine, pH 11. For flow cytometry analysis, cells were trypsinized, fixed in 75% EtOH and suspended in 700 μl of PBS containing 40 μg/ml propidium iodide and 100 μg/ml RNase. Analysis was done on a Becton Dickinson, CA Analyzer with a 20 mW argon ion laser tuned to 488 nm. Propidium iodide fluorescence was measured at 585 nm. Data were collected and analyzed with FlowJo software.

### Chromatin fractionation

Chromatin was isolated from cells suspended in buffer A (10 mM HEPES, [pH 7], 10 mM KCl, 1.5 mM MgCl_2_, 0.34 M sucrose, 10% glycerol, 1 mM DTT, protease inhibitor cocktail) containing 0.1% Triton X-100 and incubated on ice for 5 min. Nuclei were collected by low-speed centrifugation (3, 500 rpm at 4°C for 5 min), washed once in buffer A, and then lysed in buffer B (3 mM EDTA, 0.2 mM EGTA, 1 mM DTT, protease inhibitor cocktail) on ice for 30 min. Supernatants were saved (S1). Pellets (insoluble chromatin) were then collected by centrifugation of the lysed nuclei (4, 000 rpm, 4°C, 5 min); the supernatants (S2) were pooled with S1 (1:1; soluble fraction). The insoluble chromatin fractions were washed twice with buffer B, once with MNase buffer (10 mM Tris HCl, 10mM KCl, 1 mM CaCl_2_, 1 mM DTT, protease inhibitor cocktail) and centrifuged again (4,000 rpm, 4°C, 5 min). The final chromatin pellets were suspended in Laemmli buffer and heated.

For release of chromatin-bound proteins, chromatin was isolated as described above and then suspended in MNase buffer containing 2U of micrococcal nuclease (Sigma-Aldrich) and incubated on a shaker at 37°C for 20 min. The nuclease reaction was stopped by adding 0.1 mM EDTA and the mixture centrifuged (13,000 rpm, 4°C, 10 min) to obtain a supernatant (sup) containing the released proteins.

### Soft agar colony formation assay

T47D cells were transfected with Luc, Cath-D, or/and TRPS1 shRNAs using the Nucleofector Technology. Three days post-transfection, soft agar assays were performed by plating 6000 cells/well in 6-well plates. After 8 days, colonies were then stained with p-iodonitrotetrazolium violet (Sigma-Aldrich) at 37°C overnight and counted using Image J.

### Fluorescence microscopy

T47D cells grown on glass coverslips in 12-well plates were fixed with 4% paraformaldehyde, permeabilized with 0.1% Triton X100 and incubated with 2.5% goat serum to block non-specific binding (Sigma). Cells were then incubated with 10 μg/ml anti-TRPS1 goat polyclonal antibody followed by incubation with AlexaFluor 488-conjugated rabbit anti-goat IgG (1/500; Life Sciences). In other experiments, cells were first incubated with 1 μg/ml anti-BAT3 chicken polyclonal antibody (Abcam) followed by incubation with FITC-conjugated goat anti-chicken IgG (1/5000; Abcam). Cells were then washed, incubated with 2.5 μg/ml M1G8 (anti-Cath-D mouse monoclonal antibody) and then with a RITC-conjugated goat anti-mouse IgG (1/50; Invitrogen). Co-labeling by anti-TRPS1 and Cath-D antibodies was examined using a BioRad 1024 CLSM confocal microscope with a 60X (1.4NA) Nikon objective. Series of four optical sections (0.23 μm thick) were collected and projected onto a single plane. Co-staining was imaged with a 63X Plan-Apochromat objective on z stacks with a Zeiss Axioimager light microscope equipped with Apotome to eliminate out-of-focus fluorescence (slice ∼0.3 μm each).

### Microarray analysis

Gene expression patterns were examined in TRPS1, Cath-D and TRPS1/Cath-D-silenced T47D cells. Each test condition was compared to the reference condition (a pool of RNA from T47D cells transfected with Luciferase siRNA). Each experiment was repeated three times and gene expression was analyzed using Agilent^®^ SurePrint G3 Human GE 8x60K Microarrays (AMADID 28004), as specified in the manufacturer’s protocol. Test samples were labeled with Cy5 and controls with Cy3. Microarray images were analyzed with Agilent Feature Extraction (10.7.3.1), using the default settings. Microarray data were processed as follow: control probes were systematically removed and flagged probes were considered as missing values. Arrays were normalized by loess normalization, followed by quantile normalization of both Cy3 and Cy5 channels. Arrays were then normalized by quantile normalization of the M-values. A single value was computed for each transcript by taking the mean of each replicated probes. Missing values were replaced using the KNN algorithm (package ‘impute’ from R). Normalized data were then analyzed with LIMMA. The top-ranked genes were selected using the following criteria: an absolute fold-change >2 and an adjusted *p*-value (FDR) < 0.05.

### RT-qPCR

Reverse transcription of total RNA was performed at 37°C using Moloney murine leukemia virus reverse transcriptase (Invitrogen, Carlsbad, CA) and random hexanucleotide primers (Promega, Madison, WI). wReal-time quantitative PCR analyses were performed on a Light Cycler 480 SYBR Green I master and a Light Cycler 480 apparatus (both from Roche Diagnostics, Indianapolis, IN). The integrity of the PCR products was verified by melting curve analysis. Real-time qPCR values were determined by reference to a standard curve generated by RT-qPCR amplification of serially diluted cDNAs using the various primers. Quantification data were normalized to the amplification data for the reference genes encoding hypoxanthine phosphoribosyl transferase (HPRT) or ribosomal protein S9 (RPS9). The sequences of gene-specific oligonucleotides for Cath-D, TRPS1, PTHrP, ZEB2, STAT3, osteocalcin, MAOA, RBP1, RPS9 and HPRT are shown in Table S7.

### Luciferase reporter promoter assays

Cells were transfected using the Lipofectamine 2000 reagent (InVitrogen, Cergy-Pontoise, France) according to the manufacturer’s instructions (48-well plates). Transfected cells were lysed by incubation in 0.2 ml lysis buffer (25 mM Tris pH 7.8, 2 mM EDTA, 10% glycerol, 1% Triton X-100) at 4°C for 10 min. Supernatant aliquots (50 μl) were mixed with 50 μl firefly luciferase detection solution and luciferase activity measured in a luminometer (Labsystem, Les Ulis, France). Renilla luciferase activity was used to normalize transfection efficiency, according to the Promega Corporation’s instructions. Transfections were performed in triplicate. Silencing experiments linked to luciferase assays were performed using the non-specific control RNA 5′AGGUAGUGUAAUCGCCUUGdTdT 3′ (MWG Eurofins, Ebersberg, Germany).

## SUPPLEMENTARY FIGURES AND TABLES














