# Supplementary material for: Nuclear cathepsin D enhances TRPS1 transcriptional repressor function to regulate cell cycle progression and transformation in human breast cancer cells
Source: Oncotarget. 2015 Jun 27;6(29):28084–103. doi: 10.18632/oncotarget.4394 (PMC4695046; doi:10.18632/oncotarget.4394)
Supplement: Supplementary file 1 [file oncotarget-06-28084-s001.pdf]

SUPPLEMENTARY FIGURES AND TABLES

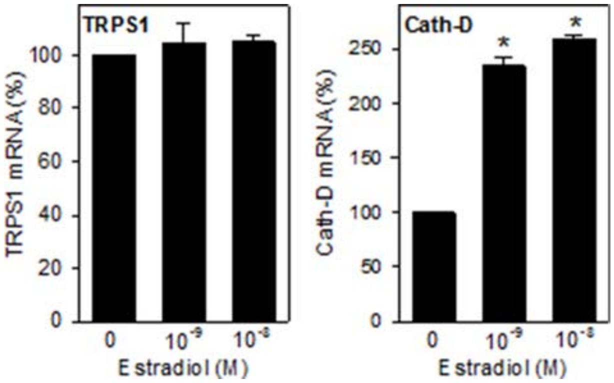

Supplementary Figure S1: Effect of estradiol on Cath-D and TRPS1 gene expression. MCF7 cells were cultured in DMEM + 10% fetal calf serum without estradiol for seven days, and then with increasing concentrations of estradiol for 48 h. Cath-D and TRPS1 mRNAs were quantified by RT-qPCR. Mean ± SD of triplicate PCR assays. *$p < 0.005$, Student's *t*-test.

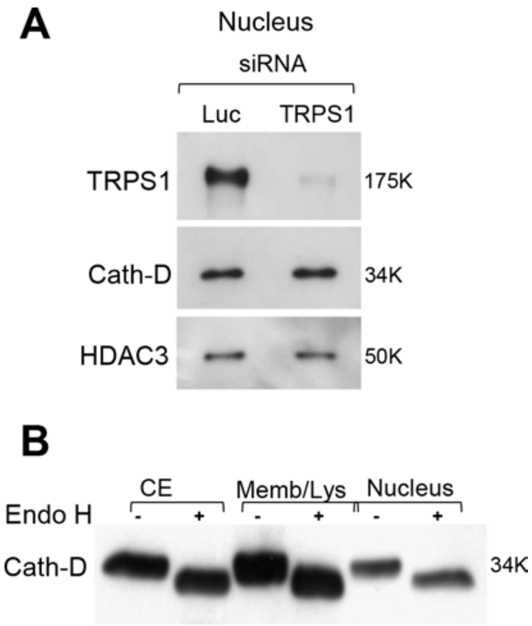

Supplementary Figure S2: TRPS1 silencing does not modify Cath-D nuclear accumulation. A. Influence of TRPS1 on nuclear Cath-D. T47D cells were transfected with Luc siRNA (20 µg) or TRPS1 siRNA2-3 (10 µg of each) and nuclear fractions were analyzed for TRPS1 and Cath-D expression by western blotting (WB) 48 h later. HDAC3, loading for nuclear fraction. **B.** Glycosylation status of nuclear Cath-D. T47D cell lysates (CE), membrane and nuclear fractions (20 µg) were incubated at 37°C with or without 25 mU endoglycosidase H overnight, and then Cath-D expression was determined by WB.

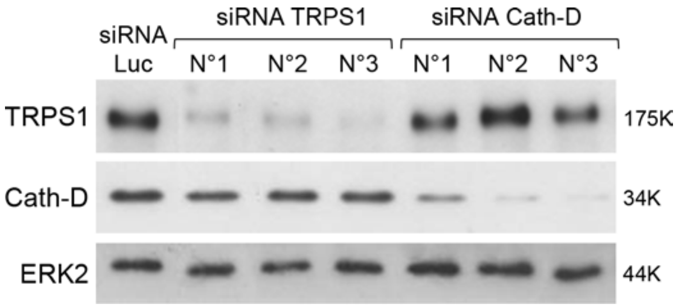

Supplementary Figure S3: TRPS1 and Cath-D silencing in ER^+ T47D cells. T47D cells were transfected with Luc siRNA (20 µg), TRPS1 siRNA1–3 or Cath-D siRNA1–3 (N°1 to 3) (20 µg of each). TRPS1 and Cath-D expression were analyzed by western blotting. ERK2: loading control.

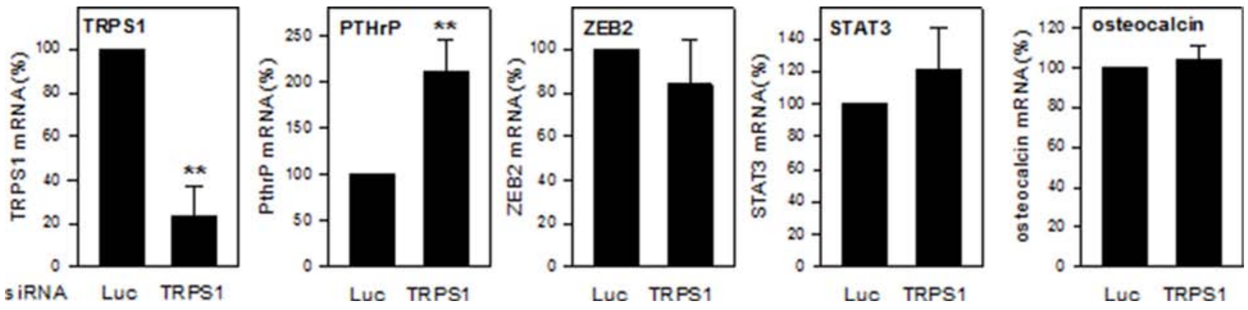

Supplementary Figure S4: Repression of *PTHrP* expression by TRPS1 in T47D cells. T47D cells were transfected in triplicate with Luc siRNA (20 µg) or TRPS1 siRNA1–3 (20 µg). After 48 h, TRPS1, PTHrP, ZEB2, STAT3 and osteocalcin mRNA levels were quantified by RT-qPCR. TRPS1 silencing did not modify ZEB2, STAT3 and osteocalcin mRNA level. Mean ± SD. $**p < 0.005$; Student's *t*-test.

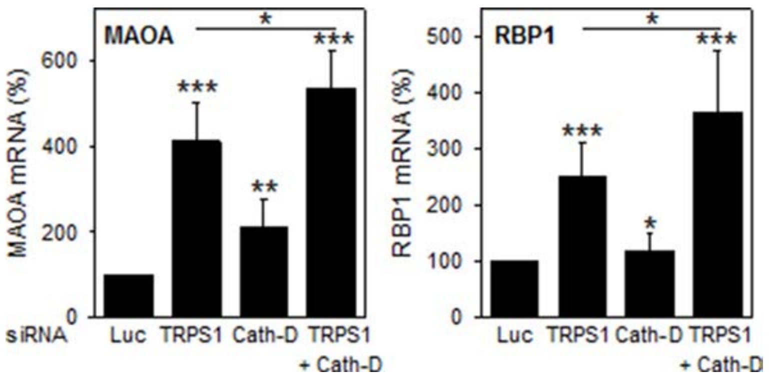

Supplementary Figure S5: TRPS1 and Cath-D co-repress *MAOA* and *RBP1* expression in T47D cells. T47D cells were transfected with Luc siRNA (20 µg), TRPS1 siRNA2–3 or Cath-D siRNA2–3 (10 µg of each), or TRPS1 + Cath-D siRNA2–3 (5 µg of each, total 20 µg). MAOA and RBP1 expression were quantified by RT-qPCR 48 h post-transfection. Mean ± SD of three independent experiments. *$p < 0.05$; **$p < 0.01$; ***$p < 0.0005$; Student's *t*-test.

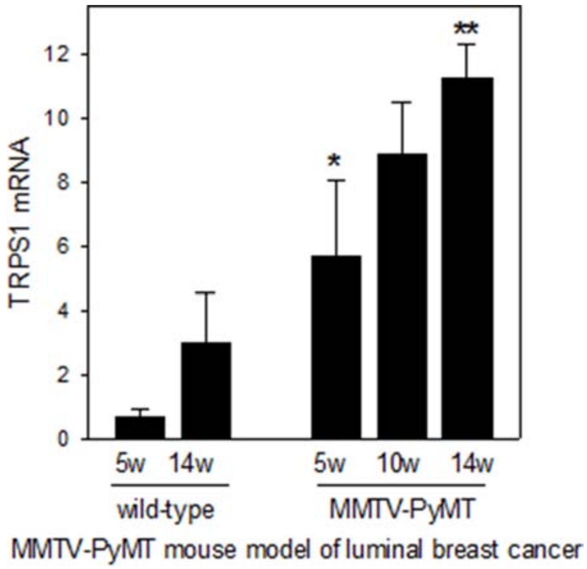

Supplementary Figure S6: TRPS1 mRNA is up-regulated in the transgenic MMTV-PyMT mouse model of ER+ luminal breast cancer. TRPS1 mRNA was quantified by RT-qPCR in mammary glands of 5- and 14-week/old wild type mice, and in mammary glands of 5-week/old (adenoma), 10-week/old (*in situ* carcinoma) and 14-week/old (late carcinoma) MMTV-PyMT transgenic mice. Mean ± SD of triplicate samples. *$p < 0.025$ *versus* 5-week/old wild type mice; **$p < 0.005$ *versus* 14-week/old wild type mice; Student's *t*-test.

Supplementary Table S1: Transcripts up-regulated in TRPS1 silenced and TRPS1/Cath-D co-silenced T47D cells. TRPS1 *versus* Ctrl: genes significantly over-expressed in TRPS1-silenced cells compared to Luc-silenced T47D cells. TRPS1-Cath-D *versus* Ctrl: genes significantly over-expressed in TRPS1/Cath-D co-silenced cells compared to Luc-silenced cells. The corresponding differences are expressed as the ratio between (TRPS1/Cath-D silencing versus Ctrl) and (TRPS1 versus Ctrl).

Supplementary Table S2: Transcripts down-regulated in TRPS1-silenced T47D cells. Genes significantly down-regulated following TRPS1 silencing: Ratio: TRPS1-silenced cells *versus* Luc-silenced cells.

Supplementary Table S3: Global transcriptome analysis of TRPS1-silenced and Cath-D-silenced T47D cells.

Supplementary Table S4: Effect of TRPS1/Cath-D co-silencing on the expression of cell cycle-related genes. Data are expressed as the fold-change (FC) of the mRNA levels of cell cycle-related genes that are significantly regulated in TRPS1-, Cath-D-, or TRPS1+Cath-D-silenced T47D cells relative to Luc-silenced cells. NA, not affected.

Supplementary Table S5: Effect of TRPS1/Cath-D co-silencing on proliferation-related genes. Data are expressed as the fold-change (FC) of the mRNA levels of cell proliferation-related genes that are significantly regulated in TRPS1-, Cath-D-, or TRPS1+Cath-D-silenced T47D cells, relative to Luc-silenced cells. NA, not affected.

Supplementary Table S6: Effect of TRPS1/Cath-D co-silencing on transformation-related genes. Data are expressed as the fold-change (FC) of the mRNA levels of genes that are significantly regulated in TRPS1-, Cath-D-, or TRPS1+Cath-D-silenced T47D cells relative to Luc-silenced cells. NA, not affected.

Supplementary Table S7: Sequences of the RT-qPCR primers, siRNAs and shRNAs

Name	Sequence
Luc siRNA	sense 5′CGUACGCGGAAUACUUCGAUU3′<br>antisense 5′UCGAAGUAUUCCGCGUACGUU3′
Non-specific control siRNA	sence 5′AGGUAGUGUAAUCGCCUUG3′<br>antisense 5′CAAGGCGAUUACACUACCU3′
Cath-D siRNA n°1	sense 5′GCUGGUGGACCAGAACAUCUU3′<br>antisense 5′GAUGUUCUGGUCCACCAGCUU3′
BAT3 siRNA n°1	sense 5′ TTTCTCCAAGAGCAGTTTA3′<br>antisens 3′TAAACTGCTCTTGGAGAAA5′
BAT3 siRNA n°2	Sense 5′GGACAAACCTGGAATTTCT3′<br>Antisens3′AGAAATTCCAGGTTTGTCC3′
Luc shRNA	5′GACTTACGCTGAGTACTTCGATCAAGAGTCGAAGTACTCAGCGTAAGTC3′
Cath-D shRNA	5′GCACAGACTCCAAGTATTACATCAAGAGTGTAATACTTGGAGTCTGTGC3′
TRPS1 shRNA	5′GGCAGGACAAGATAACAGTCATCAAGAGTGACTGTTATCTTGTCCTGCC3′
hTRPS1	forward 5′GGTCTTTGTGGTCAGGCAAT3′<br>reverse 5′CGGGAGAAAACAAGTCCAAG3′
mTRPS1	forward 5′AGGGCT TCCTGCAAGGGGCT3′<br>reverse 5′CCTCTTCGCCTCCGTAACAGCG3′
hCath-D	forward 5′TTGCTGTTTTGTTCTGTGGTTTTC3′<br>reverse 5′CAGACAGGCAGGCAGCATT3′
PTHrP	forward 5′CGGTGTTCCTGCTGAGCTAC3′<br>reverse 5′AATCTTGGATGGACTTCCCC3′
ZEB2	forward 5′GGACCAGCCACAAATGAAAG3′<br>reverse 5′GCATAAAGCCATCTTCCCAA3′
STAT3	forward 5′CCTCTGCCGGAGAAACAG3′<br>reverse 5′CTGCTCCAGGTACCGTGTGT3′
Osteocalcin	forward 5′TGAGAGCCCTCACACTCCTC3′<br>reverse 5′CCTCCTGCTTGGACACAAAG3′
MAOA	forward 5′TGGAGAATCAAGAGAAGGCG3′<br>reverse 5′CAGTCAAGAGTTTGGCAGCA3′
RBP1	forward 5′GAGGAGGATCTGACAGGCAT3′<br>reverse 5′CTCATCACCCTCGATCCACT3′
HPRT	forward 5′CTGACCTGCTGGATTACA3′<br>reverse 5′GCGACCTTGACCATCTTT3′
RPS9	forward 5′CTGCTGACGCTTGATGAGAA3′<br>reverse 5′CAGCTTCATCTTGCCCTCAT3′
